# Exposure to Waterpipe Smoke Disrupts Erythrocyte Homeostasis of BALB/c Mice

**DOI:** 10.3390/biology13060453

**Published:** 2024-06-19

**Authors:** Zannatul Ferdous, Sumaya Beegam, Nur E. Zaaba, Abderrahim Nemmar

**Affiliations:** 1 Department of Physiology, College of Medicine and Health Sciences, United Arab Emirates University, Al Ain P.O. Box 15551, United Arab Emirates; 201370004@uaeu.ac.ae (Z.F.); sumayab@uaeu.ac.ae (S.B.);; 2 Zayed Center for Health Sciences, United Arab Emirates University, Al Ain P.O. Box 15551, United Arab Emirates

**Keywords:** waterpipe smoke, erythrocytes, oxidative stress, reactive oxygen species, antioxidant enzymes, inflammation, eryptosis

## Abstract

**Simple Summary:**

Although various studies have provided evidence of a link between cardiovascular and hematological disease development and waterpipe smoking (WPS), there are a limited number of studies that have explored the impact of WPS on erythrocyte homeostasis. Here, we show that, compared with the air-exposed group, sub-chronic exposure to WPS in mice induced inflammation and oxidative stress in the plasma, decreased the number of erythrocytes and the hematocrit, and increased erythrocyte fragility. Moreover, erythrocytes in the WPS-exposed group showed increased markers of oxidative stress, ATPase activity, Ca^2+^ levels, annexin V binding, and calpain activity. In conclusion, our data demonstrate that WPS exposure elevated oxidative stress in the plasma and induced hemolysis in vivo. It also caused alterations in erythrocyte oxidative stress and eryptosis in vitro.

**Abstract:**

The prevalence of waterpipe tobacco smoking (WPS) is increasing worldwide and is relatively high among youth and young adults. It has been shown, both experimentally and clinically, that WPS exposure adversely affects the cardiovascular and hematological systems through the generation of oxidative stress and inflammation. Our study aimed to evaluate the impact of WPS exposure on erythrocytes, a major component of the hematological system, of BALB/c mice. Here, we assessed the effect of nose-only WPS exposure for four consecutive weeks on erythrocyte inflammation, oxidative stress, and eryptosis. The duration of the session was 30 min/day, 5 days/week. Control mice were exposed to air. Our results showed that the levels of C-reactive protein, lipid peroxidation (LPO), superoxide dismutase, and total nitric oxide (NO) were significantly increased in the plasma of WPS-exposed mice. The number of erythrocytes and the hematocrit were significantly decreased in WPS-exposed mice compared with the control group. Moreover, there was an increase in the erythrocyte fragility in mice exposed to WPS compared with those exposed to air. The levels of lactate dehydrogenase, LPO, reduced glutathione, catalase, and NO were significantly increased in the red blood cells (RBCs) of WPS-exposed mice. In addition, erythrocytes of the WPS-exposed group showed a significant increase in ATPase activity, Ca^2+^, annexin V binding, and calpain activity. Taken together, our findings suggest that WPS exposure elevated inflammation and oxidative stress in the plasma and induced hemolysis in vivo. It also caused alterations of RBCs oxidative stress and eryptosis in vitro. Our data confirm the detrimental impact of WPS on erythrocyte physiology.

## 1. Introduction

Waterpipe tobacco smoking (WPS), also called shisha/hookah, entails the utilization of a multi-stemmed device with water at its base, allowing the inhalation of tobacco smoke, often flavored with fruits. Toxicants found in mainstream WPS include nicotine, carbon monoxide, carcinogenic polycyclic aromatic hydrocarbons, aromatic amines, aldehydes, furanic and phenolic compounds, tar, particulate matter, heavy metals, and ammonia [1]. Numerous systematic reviews and meta-analyses have established a connection between this form of tobacco consumption and diseases commonly associated with cigarette use, including but not limited to lung cancer, oral cancer, cardiovascular disease, respiratory disease, and low birth weight [2].

A usual WPS session extends from 30 min to several hours, exposing the smoker to a smoke volume equivalent to 100–200 times that of a single cigarette [3]. Approximately 100 million people globally engage in daily WPS [4]. Among adults, the prevalence is most notable in the Eastern Mediterranean region, while among youth, the rates are significantly elevated in both the Eastern Mediterranean and European regions [2]. In the United States, the percentage of adults engaging in waterpipe smoking, defined as “current use” (smoking at least once within the past 30 days), was 9.8%, and “ever use” (smoking at any point in their lifetime) was 1.5% in 2009–2010 [5]. These numbers increased significantly to 12.3% for current use and 3.3% for ever use by 2012–2013, reflecting a similar notable rise in prevalence within the US population [6].

The diverse health effects of WPS span both acute and long-term impacts on various organ systems [4]. Both brief and extended periods of WPS have been documented to induce changes in pulmonary function. Our recent findings indicated that exposure to WPS exclusively through the nose in mice resulted in heightened airway resistance, inflammation, and oxidative stress [7,8]. Increasing evidence also suggests that WPS could induce short-term cardiovascular changes and long-term effects, potentially elevating the risk of cardiovascular disease (CVD) and triggering cardiovascular events [9,10,11]. Short-term WPS immediately increases heart rate, blood pressure, coronary blood flow, and carboxyhemoglobin (COHb) levels [12,13]. Vascular function is also affected, with increased vascular resistance and decreased blood flow, venous outflow, and venous capacitance [14]. Long-term WPS use is associated with elevated CVD risk, severity, and mortality, with potential cardiovascular effects including hypertension, hyperlipidemia, hyperglycemia, and abdominal obesity [1]. Limited data suggest a higher burden of atherosclerotic disease in this form of tobacco consumers compared to cigarette smokers [1]. Long-term WPS use is linked to a 3-fold increase in the odds of angiographically diagnosed coronary artery stenosis [1]. Alarabi et al. [15] examined the impact of WPS on platelet function, hemostasis, and thrombogenesis in mice by employing a whole-body exposure method and documented that WPS increases the risk of thrombosis, due to platelet hyperactivity. In addition, our previous data showed that WPS exposure (for 5 days and 30 days) accelerates coagulation and causes cardiac inflammation and oxidative stress in mice [16,17].

Ebrahim et al. [18] investigated the effect of WPS on hematological parameters such as RBC, hemoglobin (Hb), hematocrit (Hct), WBC, and platelet count in Wistar rats between 4 to 12 weeks of exposure, and their results indicated elevated levels of RBC, Hb, Hct, and WBC and reduced levels of platelet count. In 2012, researchers demonstrated for the first time that smoking leads to a reduction in membrane fluidity, potentially affecting the functionality of the plasma membranes in red blood cells (RBCs) among individuals with COPD [19]. The study proposed that the high content of polyunsaturated fatty acids in RBC membrane lipids makes them susceptible to hemolysis and loss of membrane stability when exposed to toxins and prooxidants present in tobacco smoke [20,21].

Although both clinical and animal studies have provided substantial evidence of a link between cardiovascular disease development and WPS (both short- and long-term use), there is a limited number of studies that have explored the impact of WPS on the RBCs/erythrocytes, a major component of the hematological system. Therefore, the goal of this study is to investigate the hemolytic effect and mechanism of action of WPS inhalation on erythrocyte inflammation, oxidative stress, and eryptosis.

## 2. Materials and Methods

### 2.1. Animals and WPS Exposure

Both male and female BALB/c mice (Animal House of the College of Medicine and Health Sciences, UAEU) were accommodated in a standard animal facility, adhering to a 12-h light-dark cycle with lights on at 6:00 am. They were placed in cages and provided with pelleted food and water ad libitum. After a one-week adaptation period, the mice were randomly divided into two groups: one exposed to air (control), and the other exposed to waterpipe smoke (WPS).

The mice were secured in gentle restraints and connected to an exposure tower following the method described by Nemmar et al. [16,17,22,23]. To expose them, a nose-only system connected to a waterpipe (InExpose System, Scireq, Montréal, Canada) was employed, and the animals were exposed through their noses. Commercially available apple-flavored tobacco (Al Fakher Tobacco Trading, Ajman, United Arab Emirates) was used, ignited with an instant light charcoal disk (Star, 3.5 cm in diameter and 1 cm in width). Similar to human usage, the smoke first passes through water before entering the exposure tower. The exposure process was managed by a computerized system (InExpose System, Scireq, Canada).

A computer-controlled puff of waterpipe smoke was administered every minute, with each puff lasting 2 s, followed by 58 s of fresh air. Each exposure session lasted for 30 min per day, 5 days a week [7,16,23]. This selection was based on work that assessed the cardiorespiratory effects of waterpipe smoke in healthy subjects [13]. The mice were exposed to either air or WPS for a duration of 4 weeks.

At the end of the exposure period, animals were sacrificed by an overdose of sodium pentobarbital and blood was collected for plasma and RBCs analysis.

This project was reviewed and approved by the Institutional Review Board of the United Arab Emirates University, College of Medicine and Health Sciences, and experiments were performed in accordance with protocols approved by the Institutional Animal Care and Research Advisory Committee (approval # ERA_2019_5983).

### 2.2. Blood Collection and Biochemical Analysis of Plasma and Erythrocytes

At the completion of the exposure period to either air or WPS, to induce anesthesia, mice were intraperitoneally injected with sodium pentobarbital (45 mg/kg), and then blood was collected from the inferior vena cava (EDTA 4%) and used for determining RBCs count by an automated cell counter (ABX Vet ABC hematology analyzer, ABX Diagnostics, Montpellier, France). The blood was then centrifuged for a duration of 15 min at a temperature of 4 °C and a speed of 900× *g*. The obtained plasma samples were kept at −80 °C awaiting analysis of C-reactive protein (CRP) concentration, which was determined using an ELISA Kit (Uscn Life Science Inc., Wuhan, China). NADPH-dependent membrane LPO was determined using a kit that measures thiobarbituric acid reactive substances (Cayman Chemical Company, Ann Arbor, MI, USA). Superoxide dismutase (SOD) was measured using a kit from Cayman Chemical Company (Ann Arbor, MI, USA). The determination of NO was performed with a total NO assay kit from R&D Systems (Minneapolis, MN, USA), which measures the more stable NO metabolites, NO_2_^−^ and NO_3_^−^ [24].

The osmotic fragility test, which evaluates the fragility of erythrocytes by subjecting them to osmotic stress, inducing lysis, was performed as described previously [25,26]. Briefly, in this test, mouse RBCs were incubated in varying concentrations of sodium chloride (NaCl), ranging from 0.1% to 0.9%, for 30 min at 37 °C. After incubation, the samples were centrifuged, and the absorbance of the supernatant was measured at 540 nm to assess the degree of hemolysis. The results, expressed as the percentage of hemolysis, were then plotted against the NaCl concentrations to generate osmotic fragility curves.

After the removal of plasma, the erythrocytes (0.5 × 10^6^ cells) were washed four times in saline (0.9%) and lysed as previously reported [27,28,29,30,31]. The samples were centrifuged at 12,000 rpm for 15 min at 4 °C and the supernatants were quickly frozen in aliquots to be used later for further analysis.

LDH activity in the collected supernatants were determined by UV assay using a commercially available kit (Roche, Basel, Switzerland). The concentrations of LPO and NO were measured as described above. Catalase was measured using a kit from Cayman Chemical Company (Ann Arbor, MI, USA). GSH was measured with a kit obtained from Sigma-Aldrich Co (St Louis, MO, USA).

Erythrocyte ATPase activity was measured using an ATPase assay kit (Abcam, Milpitas, CA, USA) as per the manufacturer’s instructions.

Intracellular calcium (Ca^2+^) levels in erythrocytes were assessed using a known method [32]. Erythrocytes were washed with 0.9% NaCl solution four times, then suspended in 1 mM Ringer’s solution, resulting in a final suspension with 0.5% hematocrit. After centrifugation at 1200× *g* for 5 min, the cells were resuspended in 5 mM Ringer’s solution with the addition of 5 μL of Fura 2AM (Calbiochem; La Jolla, CA, USA). This mixture was incubated in the dark on a shaker at 37 °C for 15 min. Following this, the cell suspensions were centrifuged at 1200× *g* for 5 min. The Fura 2AM-loaded erythrocytes were then resuspended in 1 mM Ringer’s solution and incubated for an additional 30 min at 37 °C in the dark on a shaker. Finally, Ca^2+^-dependent fluorescence intensity was measured using a fluorometer (model SFM 25, Kontron; Zurich, Switzerland) with 340 nm excitation and 510 nm emission [33].

Annexin V bound to exposed phosphatidylserine was measured using a mouse ANXA5 ELISA kit according to the manufacturer’s instructions (Elabscience, Houston, TX, USA).

The activated calpain was fluorometrically determined using a calpain activity assay kit according to the manufacturer’s instructions (Genway Biotech, San Diego, CA, USA).

## 3. Statistics

All statistical analyses were performed with GraphPad Prism Software version 7.05 (San Diego, CA, USA). Data were analyzed using the unpaired *t*-test for differences between the two groups. Data were reported as mean ± SD. *p* < 0.05 is considered significant.

## 4. Results

### 4.1. Effect of WPS on Plasma Levels of CRP, LPO, SOD, and NO

**Figure 1:**
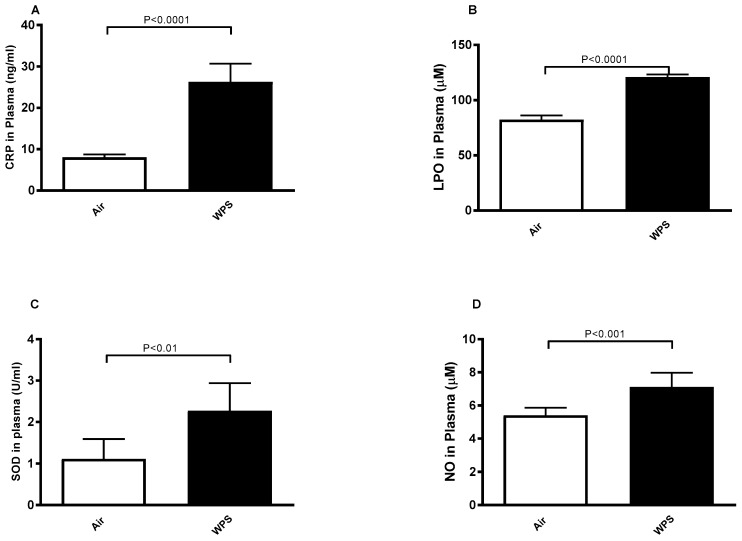
Plasma levels of C-reactive protein (CRP, (**A**)), lipid peroxidation (LPO, (**B**)), superoxide dismutase (SOD, (**C**)) and nitric oxide (NO, (**D**)) in BALB/c mice at the end of the 4-week exposure period to air (control) or waterpipe smoke (WPS). Data are mean ± SD (n = 7–8 in each group). Statistical analysis is done by unpaired *t*-test for differences between the two groups.

Figure 1 shows the effect of WPS exposure on some markers of inflammation and oxidative stress in plasma. Compared to the control group, WPS exposure significantly increased the concentration of CRP (*p* < 0.0001), LPO (*p* < 0.0001), SOD (*p* < 0.01), and NO (*p* < 0.001) (Figure 1A–D).

### 4.2. Effect of WPS on Erythrocyte Count and Hematocrit

**Figure 2:**
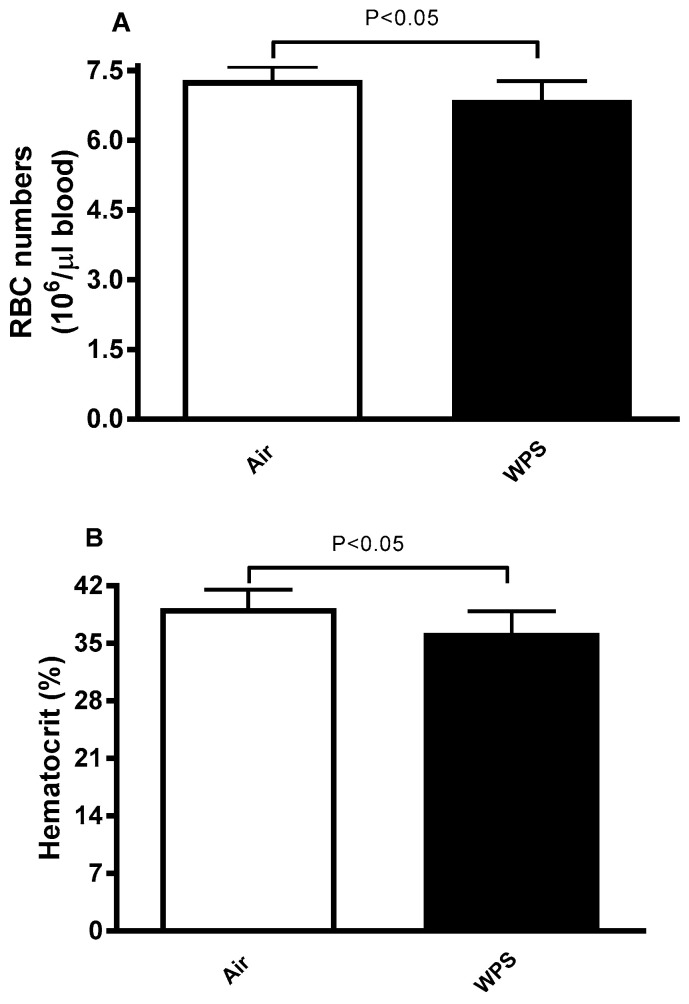
Numbers of erythrocytes (**A**) and hematocrit (**B**) of BALB/c mice at the end of the 4-week exposure period to air (control) or waterpipe smoke (WPS). Data are mean ± SD (n = 8 in each group). Statistical analysis is done by unpaired *t*-test for differences between the two groups.

Figure 2 shows that the exposure to WPS caused a mild but significant decrease in RBC numbers and hematocrit compared to controls (*p* < 0.05).

### 4.3. Effect of WPS on Red Blood Cell Osmotic Fragility

**Figure 3:**
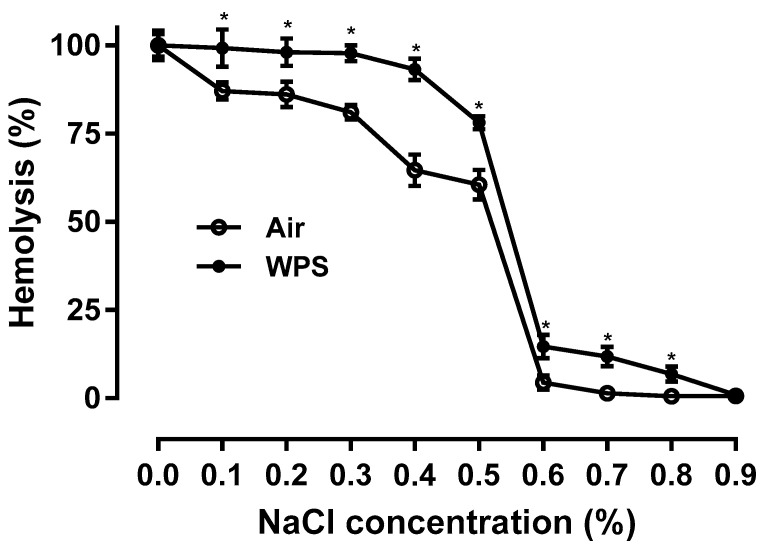
Osmotic fragility of erythrocytes obtained from BALB/c mice at the end of the 4-week exposure period to air (control) or waterpipe smoke (WPS). Data are mean ± SD (n = 8 in each group). Statistical analysis is done by unpaired *t*-test for differences between the two groups. * indicates *p* < 0.0001 compared with the control group.

Figure 3 shows the effect of WPS exposure on the osmotic fragility of erythrocytes. Compared with the control group, the erythrocytes collected from mice exposed to WPS displayed a statistically significant increase in fragility when exposed to different concentrations of NaCl (0.1 to 0.8%) (*p* < 0.0001).

### 4.4. Effect of WPS on Erythrocyte Levels of LDH, LPO, GSH, Catalase, and NO

**Figure 4:**
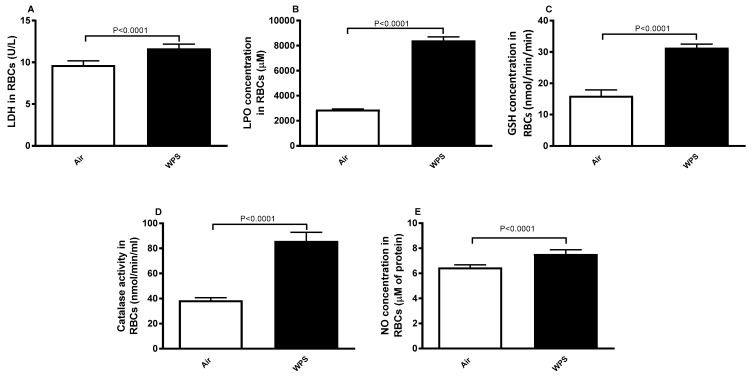
Levels of lactate dehydrogenase (LDH, (**A**)), lipid peroxidation (LPO, (**B**)), reduced glutathione (GSH, (**C**)), catalase, (**D**)) and nitric oxide (NO, (**E**)) in erythrocytes of BALB/c mice at the end of the 4-week exposure period to air (control) or waterpipe smoke (WPS). Data are mean ± SD (n = 6–8 in each group). Statistical analysis is done by unpaired *t*-test for differences between the two groups.

Figure 4A shows the effect of WPS exposure on the erythrocyte LDH activity. A significant increase (*p* < 0.0001) was observed in the WPS-exposed group compared to the control.

Concentrations of LPO, GSH, catalase, and NO are shown in Figure 4B–E. Compared with the control, the concentrations of LPO (*p* < 0.0001), GSH (*p* < 0.0001), catalase (*p* < 0.0001), and NO (*p* < 0.0001) were significantly increased in WPS-exposed BALB/c mice.

### 4.5. Effect of WPS on Erythrocyte Levels of ATPase, Intracellular Ca^2+^, Annexin V Binding, and Calpain

**Figure 5:**
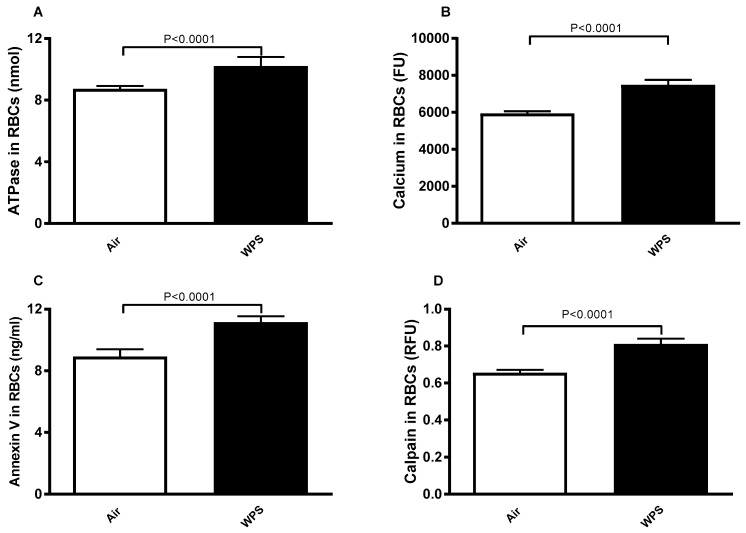
ATPase activity (**A**), calcium concentration (**B**), Annexin V concentration (**C**), and calpain activity (**D**) in erythrocytes of BALB/c mice at the end of the 4-week exposure period to air (control) or waterpipe smoke (WPS). Data are mean ± SD (n = 8 in each group). Statistical analysis is done by unpaired *t*-test for differences between the two groups.

The concentration of ATPase in erythrocytes of WPS-exposed mice is significantly elevated (*p* < 0.0001) in WPS-exposed BALB/c mice compared to the control, as shown in Figure 5A.

Figure 5B illustrates the effect of WPS exposure on cytosolic calcium concentration from Fluro3 fluorescence. The WPS exposure caused a significant increase (*p* < 0.0001) in cytosolic calcium concentration compared to the control.

Exposure of phosphatidylserine at the cell surface, a hallmark of eryptosis, was estimated from bound Annexin V. Exposure to WPS triggered Annexin V binding, as shown in Figure 5C, and there was a significant increase (*p* < 0.0001) in Annexin V binding compared to the control.

The assessment of activated calpain in the cytosol of the two tested groups is represented in Figure 5D. Compared with the control, a significant rise (*p* < 0.0001) in calpain activity was observed for erythrocytes in the WPS-exposed group.

## 5. Discussion

Our study examined the relationship between WPS exposure and the effect on erythrocytes in BALB/c mice. The results indicate altered oxidative stress markers in both the plasma and erythrocytes of WPS-exposed mice.

Experimental studies investigating tobacco exposure commonly employ two main exposure systems: nose-only and whole-body systems. In our current study, the nose-only exposure model is preferred due to its enhanced control and accuracy, mitigating issues related to rodents ingesting substances during fur cleaning. This system closely mirrors the human scenario. The 30-min exposure session in our study aligns with human studies [13,17,23]. Furthermore, our recent findings demonstrate that carboxyhemoglobin levels in mice exposed to nose-only WPS are similar to those observed in WP smokers [23].

Oxidative stress has been extensively documented as a detrimental condition associated with the development of severe diseases such as cardiovascular diseases and cancer [34]. It occurs due to an imbalance between free radical formation and antioxidant defense, particularly when reactive oxygen species (ROS) concentration overwhelms the antioxidant capacity within the erythrocytes [35]. Existing literature, spanning human, animal, and in vitro models, robustly supports the assertion that tobacco smoking induces oxidative stress [36,37,38,39,40,41,42,43]. Animal models reveal that exposure to WPS results in oxidative damage, inflammation, subsequent tissue injury, and oxidative DNA damage across various organs, including the lungs, heart, brain, kidney, liver, and reproductive organs [36,37,38,39,40,41,42,43]. Human studies further demonstrate an increase in DNA oxidation, lipid peroxidation, and alterations in catalase, SOD, and GSH in various body fluids and tissues of tobacco smokers compared to non-smokers [43,44,45]. Although systematic reviews conducted by Waziry et al. [11], El-Zaatari et al. [4], and Haddad et al. [46] elaborate upon the health impact of WPS on various organ systems, there is limited information available regarding the relationship between WPS-induced oxidative stress and erythrocytes.

In the circulating bloodstream, erythrocytes are highly susceptible to oxidative processes due to continuous exposure to high oxygen levels, making hemoglobin prone to autoxidation and providing an endogenous source of ROS in erythrocytes [35]. Exogenous ROS, released primarily from activated inflammatory cells such as macrophages, neutrophils, and endothelial cells, can cause oxidative stress to erythrocytes by diffusing and crossing the membrane [47]. Prolonged exposure of RBCs to ROS can lead to cellular damage, encompassing oxidation of lipids and proteins and resulting in impairments to enzymes and ion transport proteins [48]. Moreover, ROS can impact erythrocyte proteins by oxidizing the protein backbone, inducing cross-linking or oxidizing amino acids. Moreover, ROS causes cleavage of polyunsaturated fatty acids at their double bonds, leading to the formation of malondialdehyde, the main end product of LPO, which further reacts with lipids and proteins of the membrane [48]. In conjunction with other ROS, NO, a marker of nitrosative stress, increases in states of vascular inflammation and oxidative stress [49]. Another sensitive marker of inflammation, CRP, present in the vessel wall, is reported to be associated with endothelial cell dysfunction and the progression of atherosclerosis [50].

In the present study, we noted a slight but significant reduction of RBCs count and hematocrit in the WPS-exposed group compared with the control group, indicating the occurrence of hemolysis. Moreover, we showed an increase in the osmotic fragility of erythrocytes collected from WPS-exposed mice. Previous studies have demonstrated that smokers have significantly lower RBC counts compared to nonsmokers, with these reductions varying according to the intensity of smoking [21,51].

While comparing the oxidative and erythrocyte antioxidant defense status among the two studied groups (WPS and control), our current data clearly demonstrates a significant rise in CRP, LPO, SOD, and NO in plasma, and a rise in LPO, GSH, catalase, and NO in RBCs. Unlike certain cells, erythrocytes lack the ability to repair damaged components through resynthesis. Therefore, they depend entirely on enzymatic antioxidants, including SOD, catalase, and glutathione peroxidase, and nonenzymatic antioxidants such as GSH, throughout their 120-day lifespan [52,53]. These enzymatic and non-enzymatic antioxidants collectively establish a robust defense system crucial for detoxifying the cell from oxygen-free radicals. For instance, SOD provides the first line of defense against free radicals. It is a cytosolic, copper-zinc-containing enzyme that catalyzes the conversion of superoxide radicals to the less reactive hydrogen peroxide, which is further decomposed by catalase and GSH to O_2_ [53]. Reduced GSH contains the sulfur-containing amino acid cystine, a rate-limiting amino acid in GSH biosynthesis [48]. The presence of cystine contributes to sustaining the reduced status of the non-protein sulfhydryl group of the cell membrane. Likewise, catalase is involved in the conversion of H_2_O_2_ to H_2_O and plays a shared role in H_2_O_2_ detoxification [48]. Moreover, NO is an efficient free radical scavenger [54]. Hence, alteration of these indices in our current study potentially indicates oxidative damage and compensatory antioxidant response of erythrocytes induced by WPS. These data seem to be in line with previous studies, which found elevated LPO, GSH, catalase, and nitrite/nitrate levels in the plasma and RBCs of moderate smokers and concluded that increased antioxidant status protects RBCs from free radical damage induced by cigarette smoke [28,55,56,57]. On the contrary, Orhan et al. [58] reported a significant decrease in SOD and GPx activities in erythrocytes of smokers as compared to nonsmokers, suggesting that smoking-associated ROS leads to saturation of these antioxidant enzymes, hence reducing their bioavailability. This variation could be attributed to the severity and type of tobacco being smoked.

LDH is an essential enzyme in the cellular energy production process from glucose, making it an effective indicator of irreversible cell damage. Thus, cell membrane integrity is often measured by LDH activity in the cell medium [59]. It is well known that LDH is involved in the conversion of lactate to pyruvate intracellularly. Increased LDH levels within the red blood cells can occur due to several conditions causing tissue hypoxia, such as anemia [59]. Accordingly, the increase in intracellular LDH observed in the present work could be related to the hypoxia induced by WPS exposure [23,60]. Additionally, in our experimental setup, we measured the intracellular LDH following the in vitro lysis of the erythrocytes. Consequently, since erythrocytes from WPS-exposed mice had increased membrane fragility, they likely released LDH more effectively than those from the control group. To further evaluate cell membrane function, we assessed the ATPase activity in erythrocytes of the experimental groups. ATPases are widely recognized as molecular motors that harness the energy derived from ATP hydrolysis to drive various cellular processes, including cellular metabolism, protein assembly and trafficking, replication, transcription, and ion pumping [61]. The ATPase assay serves as an indirect measure of the activity of the efflux transporter, which in turn is responsible for mediating the transport of substrates across cell membranes against a concentration gradient. Impairment of ATPase activity in human erythrocytes and platelets of chronic smokers have been previously reported, in addition to altered membrane properties and a decrease in membrane fluidity [62,63]. In contrast, our current data reveal increased total ATPase activity in RBCs of WPS-exposed animals compared to a control, suggesting a potential role in maintaining physiological intracellular ion levels and essential transmembrane gradients.

Mature erythrocytes, being highly specialized cells, lack normal cell organelles, such as nuclei and mitochondria, which are vital for the regulation of apoptosis, an innate mechanism of programmed cell death [64]. The senescence involved in mature erythrocytes is termed eryptosis, characterized by distinct changes in shape and plasma membrane, with translocation of phosphatidylserine from the inner leaflet of the cell membrane [65]. Exposure to oxidative stress triggers erythrocytes to generate molecular signals, activating Ca^2+^ permeable cation channels and leading to increased Ca^2+^ entry into the cells. The subsequent activation of Ca^2+^-sensitive K+ channels results in cell shrinkage and scrambling of the erythrocyte membrane, exposing phosphatidylserine at the cell surface [66,67]. The changes in membrane protein and lipids contribute to functional and structural alterations, resulting in decreased membrane stability and premature erythrocyte removal [48].

Eryptosis can be triggered by oxidative stress and has been associated with chronic inflammatory disease, including atherosclerosis [65]. Additionally, it has been shown to contribute to heart failure-associated anemia, driven by oxidative stress, energy depletion, and osmotic imbalance [68]. Earlier research indicates that eryptosis increases the risk of thrombosis and cardiovascular disease, particularly in individuals who are overweight or obese [69,70]. Eryptotic erythrocytes may impair microcirculation by adhering to vascular endothelial cells, leading to obstruction and various cardiovascular side effects in addition to anemia [69,70]. While the cytotoxic impact of smoking on erythrocytes is well recognized, there is relatively scant information concerning the connection between smoking and eryptosis. Limited in vitro evidence suggests that exposure of erythrocytes to cigarette smoke extract of CO can induce eryptosis [71,72]. Additionally, increased eryptosis was observed by Attanzio et al. [73] in a small-scale study that compared 21 healthy male smokers to 21 non-smokers. Hence, to further evaluate functional and structural alterations of eryptosis following WPS exposure, we conducted assessments on intracellular Ca^2+^ concentration, annexin V binding, and calpain activity. Typical features of eryptosis including cell shrinkage, membrane blebbing, and phosphatidylserine externalization have been demonstrated in erythrocytes exposed to Ca^2+^ ionophore ionomycin or A23187 [65]. Annexin V is a Ca^2+^-dependent cellular protein that has the ability to bind with phosphatidylserine and is used as an apoptotic marker when exposed to the outer leaflet of the cell membrane [65]. Unlike apoptosis of erythroblasts, which is caspase-dependent, the mature erythrocyte is driven into eryptosis by an increase in intracellular calcium, which in turn triggers activation of calpain [66]. Calpain, a calcium-dependent cystine protease, similar to caspases, exists as a proenzyme and is reported to play a role in eryptosis [67]. Our present study data show a significant increase of intracellular Ca^2+^, annexin V binding, and calpain activity in RBCs of WPS-exposed animals compared to a control. Our results seem to validate the association between erythrocyte damage induced by oxidative stress, elevated cytosolic calcium levels, and the subsequent rise in calpain activity.

To conclude, the impact of in vivo exposure of BALB/c mice to WPS was evaluated on erythrocytes. The core findings in our study demonstrated increased erythrocyte lipid peroxidation, antioxidant status, and NO, with a decrease in the RBC count or hemolysis of WPS-exposed mice. Our results potentially suggest that the antioxidant enzymes and NO are quenching free radicals produced by exposure to WPS. Additionally, we showed WPS exposure increased cytosolic Ca^2+^_,_ annexin V binding, and calpain activity, highlighting the underlying mechanism involved in oxidative stress-induced eryptosis. Moreover, we found that a high proportion of eryptosis in WPS-exposed mice was associated with a reduced number of erythrocytes and an increase in osmotic fragility. Further research is warranted to comprehensively evaluate the cardiovascular risk burden associated with WPS and its impact on long-term mortality. Furthermore, in vitro experiments using isolated erythrocytes incubated with WPS should be performed to understand the potential of antioxidant molecules in neutralizing oxidative damage.

## Data Availability

The data presented in this study are available upon reasonable request from the corresponding author (A.N.).
